# Current Evidence Regarding Shoulder Instability in the Paediatric and Adolescent Population

**DOI:** 10.3390/jcm13030724

**Published:** 2024-01-26

**Authors:** Aziz Rawal, Franziska Eckers, Olivia S. H. Lee, Bettina Hochreiter, Kemble K. Wang, Eugene T. Ek

**Affiliations:** 1 Melbourne Orthopaedic Group, Windsor, Melbourne, VIC 3181, Australia; aziz.rawal@hotmail.com (A.R.); franziska.eckers@googlemail.com (F.E.);; 2 Orthopädie und Traumatologie, Universitätsspital Basel, 4031 Basel, Switzerland; 3 Victorian Paediatric Rehabilitation Service, The Royal Children’s Hospital, Melbourne, VIC 3052, Australia; oliviashienhui.lee@rch.org.au; 4 Department of Orthopaedics, Balgrist University Hospital, University of Zurich, 8006 Zürich, Switzerland; 5 Department of Orthopaedic Surgery, The Royal Children’s Hospital, Melbourne, VIC 3052, Australia; kemble.wang@rch.org.au; 6 Department of Surgery, Monash University, Melbourne, VIC 3800, Australia

**Keywords:** shoulder instability, shoulder dislocation, paediatric, adolescent, traumatic instability, atraumatic instability, multidirectional instability, muscle patterning

## Abstract

Paediatric and adolescent shoulder instability is caused by a unique combination of traumatic factors, ligamentous laxity, and pattern of muscle contractility. The multifactorial nature of its aetiology makes interpretation of the literature difficult as nomenclature is also highly variable. The purpose of this review is to summarize the existing literature and shed light on the nuances of paediatric and adolescent shoulder instability. The epidemiology, clinical features, imaging, and management of all forms of paediatric shoulder instability are presented. The main findings of this review are that structural abnormalities following a dislocation are uncommon in pre-pubertal paediatric patients. Young post-pubertal adolescents are at the highest risk of failure of non-operative management in the setting of traumatic instability with structural abnormality, and early stabilisation should be considered for these patients. Remplissage and the Latarjet procedure are safe treatment options for adolescents at high risk of recurrence, but the side-effect profile should be carefully considered. Patients who suffer from instability due to generalized ligamentous laxity benefit from a structured, long-term physiotherapy regimen, with surgery in the form of arthroscopic plication as a viable last resort. Those who suffer from a predominantly muscle patterning pathology do not benefit from surgery and require focus on regaining neuromuscular control.

## 1. Introduction

Shoulder dislocation and recurrent instability is increasingly common in the paediatric and adolescent population [1]. Possible causes include higher levels of competitive sports at younger ages, and better recognition and diagnosis of instability episodes. Children are more likely to risk injury in pursuit of their athletic goals, whether those be elite aspirations, scholarships, or leisure [1].

Shoulder instability can be defined as undesirable translation of the humeral head in the glenoid fossa leading to pain or discomfort [2]. It can occur after structural damage to the glenohumeral joint following traumatic dislocations or repetitive microtrauma, or be attributable to ligamentous laxity and/or abnormal muscle patterning [3,4]. The underlying cause of each presentation is often multifactorial and can make management challenging. Interpretation of the existing literature is also challenging, as the definition of different forms of instability varies, as does, sometimes, the nomenclature itself. Furthermore, as the physiology of paediatric patients changes with age, variations in age range included for studies assessed make subgroup analysis difficult.

The focus in the majority of the literature is on traumatic shoulder instability, where post-pubertal adolescents (14 to 16 years of age) are known to have one of the highest incidences of instability and failure of treatment, whether operative or non-operative [5,6,7]. Early stabilization should be considered, and augmentations to standard labral repair such as the remplissage procedure have shown added benefits [8]. The Latarjet procedure can also be used in higher risk patients, but its complications can carry greater morbidity in a younger patient [9]. Although re-dislocation is rarer in the younger, skeletally immature patient (aged less than 14 years of age), when structural damage occurs, failure of non-operative management remains high [10,11].

For patients with multi-directional shoulder instability attributable to generalized ligamentous laxity, evidence is weak due to the lack of studies that isolate patients with generalized laxity only. Within these limits, a long-term structured specialized physiotherapy program is the mainstay of treatment, and arthroscopic plication can be considered if non-operative means fail [12,13,14]. Shoulder instability attributable to abnormal muscle patterning remains poorly understood. There are no high-quality studies evaluating best management for this subgroup, in part due to the rarity of the pathology [15]. As the focus of management is on regaining neuromuscular control, there have been promising recent developments in the field, including the utility of a shoulder pacemaker device to augment physiotherapy regimens [16].

A narrative review is important in this broad and heterogenic topic to analyse the existing literature for clinicians to gain an in-depth understanding of how shoulder instability in young populations differs from that in adults, how our understanding has evolved, how to best identify the different forms of instability, and how to best treat them. Prior literature reviews have focused on traumatic instability, whereas this in-depth summary includes the results of the few systematic reviews and meta-analyses addressing subtypes of paediatric and adolescent instability [17,18,19,20,21]. For the purposes of this review, the terms “children” and “paediatric” population will refer to patients aged 17 years and under, whereas the term “adolescents” refers to patients who are 10 to 19 years of age.

## 2. Patho-Anatomy

Following a traumatic instability event, several structures in the glenohumeral joint can be damaged, leading to recurrent instability. This occurs in both adolescents and adults in a similar fashion. In an anterior instability event, the capsulolabral complex is often damaged, resulting in a Bankart lesion at the anterior–inferior glenoid labrum [22]. Anterior labroligamentous periosteal sleeve avulsion (ALPSA) and glenolabral articular disruption (GLAD) may also occur [23,24]. The inferior glenohumeral ligament (IGHL) may avulse from the humerus resulting in a humeral avulsion of the glenohumeral ligament lesion (HAGL) [25]. In terms of bony architecture, the anterior glenoid may be fractured or deficient following an anterior instability event, and there may be a depression in the posterosuperior humerus caused by impaction of the humerus against the anterior glenoid resulting in a Hill-Sachs lesion (HSL) [26,27]. In posterior traumatic instability events, the reverse may happen. Reverse Bankart lesions occur at the posterior labrum. Kim’s lesions are superficial tears at the junction between the posterior glenoid cartilage and labrum [28]. Reverse HSLs are located at the anteromedial humerus and reverse HAGL are posterior avulsions of the glenohumeral ligaments off the humerus [29,30]. However, compared to their anterior–inferior counterparts, posterior subluxations or dislocations usually occur directly posterior.

Multidirectional instability (MDI) is most often described as instability in two or more planes of motion [31]. It may occur due to structural abnormalities following trauma or microtrauma, such as those seen in swimmers or throwing athletes, or be related to generalised ligamentous laxity disorders such as Ehlers–Danlos (EDS) or Marfan’s syndrome [31]. Children are more likely to be lax than adults due to the nature of their collagen composition [32]. Type I collagen takes over from the far more elastic type III collagen as a person ages. Patients with MDI may possess higher levels of type III collagen [33]. Although stretchier, children are not as likely to suffer structural damage to their tissue due to there being less chance of permanent plastic deformation [10].

Muscle patterning issues contributing to shoulder instability occur due to disorganisation of the normal recruitment of muscles around the glenohumeral and scapulothoracic joints [34]. Aberrant muscle contractions and an imbalance of over- and under-active muscles may result in instability or frank dislocations [34]. It may also be associated with scapular dyskinesia, leading to abnormal scapula posturing and position of the glenohumeral joint [15].

The Stanmore classification system groups shoulder instability into three poles: traumatic (type I), atraumatic (type II), and muscle patterning (type III). Children and adolescents are less likely to have a predominantly type I instability pattern and are more likely to have a combination of the three aetiologies [3].

## 3. Epidemiology

Most epidemiological data on paediatric shoulder instability refers to traumatic shoulder instability, possibly neglecting the different forms of instability. Several studies aim to quantify the incidence (Table 1). In general, the peak at-risk period for dislocation in adolescence appears to be near the age of skeletal maturity. In this age group, patients are also at the highest risk of re-dislocation. In contrast, dislocations and subsequent recurrent instability are rare in the pre-pubertal population [5,35,36,37].

In keeping with these findings, Old’s systematic review of the literature prior to 2015 found that 14–18-year-old patients were 24 times more likely to experience re-dislocation than those aged 13 years and below, and those with a closed physis were 14 times more likely to dislocate than those with an open physis [18]. When comparing adolescents with adults, Hovelius’ long-term study of shoulder dislocations found the risk of recurrence was twice as high in the 12–20 age group when compared to the 20–29 age group [38].

## 4. Diagnosis

### 4.1. History and Clinical Examination

A thorough history should be taken to assess for pain, mechanism of injury, prior instability events, and other associated symptoms. In the setting of MDI, young patients can present with atypical symptoms, such as neuropathic symptoms or clicking [39]. Patients may describe ‘clunks’, which can be voluntary or involuntary and indicate a muscle patterning element [15].

In an outpatient setting, common provocative tests for the assessment of anterior instability include the load and shift test, the anterior apprehension test, and the relocation sign [40,41,42]. A positive Jerk test, Kim test, or push–pull test may suggest posterior instability [43,44].

Sulcus and Gagey signs can suggest inferior instability or capsular laxity and are useful for evaluating MDI [45]. The Beighton score for generalised ligamentous laxity should be assessed. A score of over five out of nine should raise the suspicion of ligamentous laxity [46].

As muscle patterning instability can be caused by muscular imbalances, the strength of the rotator cuff, periscapular, and core stabilising muscles should be assessed. As scapula dyskinesia is thought to be a risk factor, and scapulothoracic abnormal motion should also be assessed for [15].

### 4.2. Imaging

#### 4.2.1. X-ray

Standard plain X-rays include AP, scapula Y views, and axillary views. Further useful views include the Westpoint view for glenoid bone loss (GBL) and the Stryker notch view for Hill-Sachs deformity. Care should be taken in assessing for the presence of subtle glenoid rim fractures [47]. A high and flat acromion, seen on the scapula Y view, has been shown to be associated with posterior instability in adults and may also be valid in children and adolescents [48,49].

As can occur in adults, paediatric traumatic shoulder dislocation can be associated with greater or lesser tuberosity fractures of the humerus. Open physes in the shoulder (e.g., coracoid base and glenoid physes) can be mistaken for fractures.

#### 4.2.2. Cross-Sectional Imaging

A CT provides excellent assessment of bony architecture, avulsion fracture, glenoid rim fractures, and bone loss. Its use in the paediatric population should be balanced against the potential effects of radiation.

MRI is important for assessing injury to the joint capsule, glenohumeral ligaments, labrum, and cartilage. Fat suppressed imaging will help identify bony oedema and provide insight into the pattern of injury. MRI is generally useful and recommended after most first-time dislocations. MRI can be helpful in differentiating between MDI, muscle-patterning problems, and true traumatic instability with structural damage.

##### Bone Loss

The same CT measurements for assessing GBL, humeral bone loss, and combined bone loss in adults may also be similarly relevant in the paediatric population. Some studies have demonstrated that MRI can be a reasonable alternative to a CT to evaluate bone loss and characterise off-track lesions [50,51,52]. The lack of radiation with MRI may be of more value in the paediatric population. However, there is a lack of literature validating these measurements specific to the paediatric population.

The Glenoid index (glenoid height to width ratio) can also be measured using MRI. Anterior instability is associated with tall and narrow glenoids. Yellin et al. confirmed this in a paediatric population. In their study, a GI ≥ 1.45 was 83% sensitive and 79% specific for predicting dislocation [53]. Despite this, when specific measurements of bone loss are needed CT remains superior. The measurement of glenoid height and width on a 2D MRI vs. a 3D CT shows the CT to be more precise with respect to inter- and intra-rater agreement when measured by a musculoskeletal radiologist and an orthopaedic trainee [54]. Three-dimensional CT reformats are also recommended over 2D CT slices for HSL defect measurements, as 2D measurements have a high error rate and lower inter-observer reliability when measured by two orthopaedic surgeons and one non-medical observer [55].

When interpreting MRIs in the paediatric population, location and presence of ossification centres and physes should also be carefully considered. These can be mistaken for glenoid bone injuries, particularly the anterior glenoid ossification centre.

##### MDI

**Figure 1:**
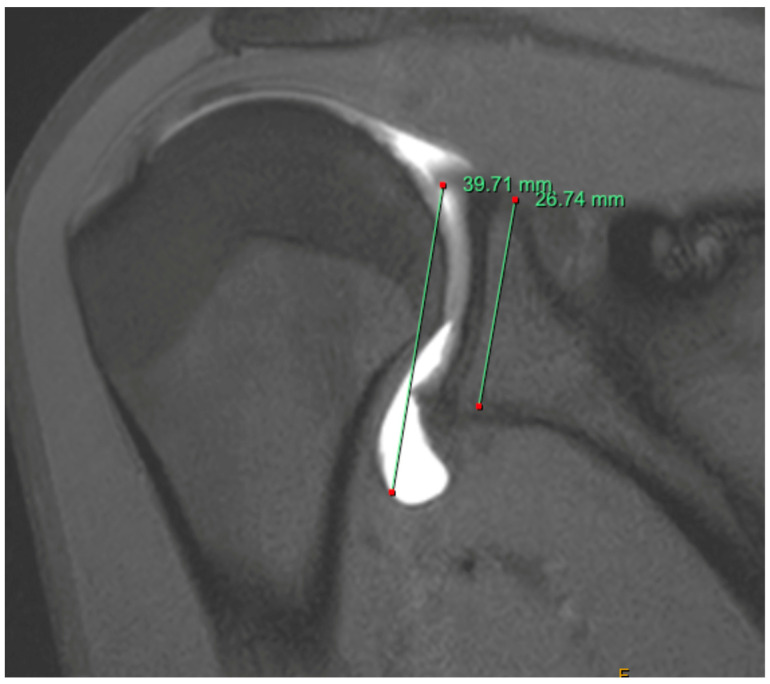
MR arthrogram of patient with symptomatic MDI. The gleno-capsular ratio is recorded as 1.48.

MR arthrogram can be useful for the assessment of possible MDI. The gleno-capsular ratio (GC) can be assessed on an arthrogram by measuring the distance between the most superior aspect of the glenoid and the most inferior aspect of the capsule and dividing that by the distance between the most superior aspect of the glenoid and the most inferior aspect of the glenoid (Figure 1) [56]. It is an indicator of the size of the inferior capsule. MDI should be suspected in patients with a GC > 1.42 with a sensitivity and specificity of 92.3 and 89.2%, respectively [56]. An inferior labro-capsular distance, or the distance from the inferior glenoid to the inferior capsule, of over 16.88 mm can also be used to screen for MDI on an arthrogram with a sensitivity of 77% and a specificity of 96% [57]. Rotator intervals with a width greater than 15.2 mm and a depth greater than 6.4 mm may also suggest MDI, with a sensitivity and specificity of 81% and 92%, respectively [58].

### 4.3. Proprioceptive Testing

As the shoulder joint is inherently unstable, it relies heavily on neuromuscular control and proprioceptive awareness to prevent injury. Proprioceptive deficits have been linked to shoulder injury, recurrence of injury, and persistence of disability [59,60]. Bilateral shoulder proprioception deficits have even been shown in patients with unilateral shoulder instability [61]. Efforts have, therefore, been made to measure proprioception objectively and to manufacture rehabilitation programs to improve joint position sense (JPS) and kinaesthesia, however, there is no universally accepted method of quantifying proprioceptive defects at the shoulder. Ager’s systematic review revealed that a passive protocol assessing JPS or a detection of movement protocol (threshold to detection of passive movement) employing external rotation and internal rotation at 90 degrees of shoulder abduction are the most reliable for assessing proprioception, and that the isokinetic dynamometer has the highest reliability measures [62]. Despite promising advancements, the utility of proprioception evaluation is limited in a clinical setting due to the complexity and intricacies of the electronic custom-made systems [62]. More needs to be understood about proprioception’s role and exact mechanisms at the shoulder for it to influence treatment decision making and be used to assess rehabilitation success [62].

## 5. Management of Traumatic Instability

Recurrent instability can potentially lead to irreversible chondral damage of the glenohumeral joint. A study analysing 282 shoulders in patients below 40 years of age found that patients with arthritis were far more likely to have had multiple episodes of instability [63,64]. The goal of treatment is, therefore, to address pain and instability, prevent recurrent dislocation, and to protect the shoulder long term.

### 5.1. Non-Operative Management

Following an anterior dislocation, both the position and duration of immobilisation are subject to discussion, with no clear benefit in immobilizing for greater than one week, or with any brace other than the standard sling [65,66]. A physiotherapy program may involve strengthening of the periscapular and rotator cuff muscles and improving humeroscapular coordination to gain stability at the glenohumeral joint until the patient satisfies return to sport criteria [67]. These programs vary significantly if features of the Stanmore type II or III instability are also present [3].

#### Outcomes of Non-Operative Management

Some studies suggest that non-operative management may have greater success in skeletally immature patients [18,35]. This is possibly due to a lower rate of structural damage following a dislocation event, and the fact that these events are likely the result of a combination of type I, II, and III instability. In Cordischi’s series of 10–13-year-olds with first-time traumatic instability, the re-dislocation rate was 21% and those that did re-dislocate had a concomitant HAGL lesion [11]. Lampert found that patients older than 14 years of age re-dislocate at a very high rate (27 of their 28 patients who underwent conservative management failed) and of those below 14 years of age, 0% of their cohort re-dislocated after non-operative management alone [68]. Deitch suggested that surgical stabilisation in patients aged 11 to 18 years following first-time dislocation is not beneficial as the non-operative and operative groups had similar recurrence rates. However, there was no subgroup analysis of skeletally immature patients, and no routine MRI following initial dislocation to assess for the presence of structural damage [69]. In Postachini’s long-term follow-up study, there was a 33% rate of re-dislocation in the under-13s group compared to 90 percent in the 14–17-year-old group. Structural injuries such as Bankart lesions were only found in the older group [10]. Thus, the literature would strongly suggest the presence of structural damage shown on an MRI following initial instability much better predicts the risk of re-dislocation rather than age.

For the adolescent population, recent research has shown that the younger the adolescent, the higher the likelihood of recurrent instability following non-operative management. Leland found that for every year of decrease in age at initial instability, with a child aged 14 years being the youngest in their cohort, the risk of recurrent instability and surgical intervention increased by 4.1% and 2.8%, respectively [6]. Gigis found that non-operative management had poor results in adolescents aged 15 to 18 years following first-time dislocation. A total of 70% of patients re-dislocated in the non-operative group compared to 13% who underwent early stabilization [7].

Although more likely to fail, initial non-operative treatment is still used in high-risk collision athletes to allow a return to in-season competition. High school rugby players in Japan have been found to have a re-dislocation rate of 54.3% following non-operative management [70]. In American high school football players, 87% of athletes return to sport (RTS) following first-time dislocation, but with high rates of re-dislocation (40/97). Bracing was found to confer no benefit in terms of RTS or preventing instability events [71]. In Tokish’s study validating a Non-Operative Instability Severity Index Score (NISIS) to discern when non-operative management is viable, 79% of the cohort of high school patients returned to sport. Of the patients who had a NISIS score above seven, 97% were able to complete for an entire subsequent competitive season of sport without a time loss event due to shoulder issues [72].

Overall, the literature would suggest that recurrent dislocations are uncommon below 13 years of age. However, the type of instability is unclear in this age group: it is unclear whether there may be predominant type II or III instability and whether there had been MRI-documented structural injury in the shoulder. When structural injury does exist on an MRI, even if the age is very young, risk of re-dislocation appears to be high. Possible explanations include less constraint on the more supple and elastic tissue, poorer compliance to rehabilitation, poorer muscle bulk, and higher activity levels.

### 5.2. Surgical Stabilisation—Bankart and Labral Repairs

Surgical management of instability can be open or arthroscopic, can involve repair of the capsulo-gleno-labral complex, or involve bony augmentation. In the paediatric and adolescent population, arthroscopic repair of the gleno-labral complex is the most common treatment for traumatic instability with structural injuries [19]. In a prospective review by Ozturk on return to sport following arthroscopic Bankart repair, which included adolescents as young as 12 years of age, there was a 13% failure rate at two years and 87% of patients were able to return to play. Those with a HSL, generalised laxity, and greater than five dislocations had higher recurrence rates. There were also no significant differences between the adolescent group and the 20 to 25 years of age group [73]. A recent systematic review also has a favourable return to sport rate of 95% amongst adolescents [19].

Regarding open Bankart repair, Hickey et al. reported a 24% re-dislocation rate in patients aged 15–18 years and 90% of these patients were able to reach their pre-injury level of sport, which is not dissimilar to the success rate for arthroscopic repair [74]. Shymon et al. also showed no significant differences between the open and arthroscopic approach in adolescents, concluding it remains a valid surgical option [75].

In contrast, other studies have quoted unacceptable recurrence rates post arthroscopic gleno-labral repair surgery. Torrance looked at recurrence amongst rugby and other collision athletes aged 14–17 years who underwent arthroscopic stabilisation and found that 51% re-dislocated following further traumatic sporting injuries [76]. Athletes younger than 16 years of age were also 2.2 times more likely to suffer a recurrence. Interestingly, subcritical levels of bone loss (HSL < 25% and GBL < 20%) did not correlate with higher failure rates, which was not the case in larger meta-analyses [20]. Nixon’s case series revealed a recurrence rate of 31% following arthroscopic stabilisation in patients aged 11 to 18 years, but unfortunately could not ascertain any statistically significant risk factors within this age group.

Reported risk factors for failure as identified in a recent meta-analysis include adolescence, GBL, HSL, ALPSA lesions, collision sports, delay to surgery, and multiple dislocations [20]. In a large case-control study of patients <18 years of age, Cheng found that risk factors of recurrence following arthroscopic Bankart repair include glenoid bone loss, glenoid retroversion < 6°, multiple dislocations, and open physes [77]. The reasons for a higher recurrence rate following surgery in younger adolescents is not fully clarified. Possible factors include higher elasticity of tissue, higher activity level, and poorer compliance with rehabilitation [9]. Uninsured patients with reduced access to care are also correlated with the development of bone loss and a higher likelihood of recurrence, possibly due to delays to care and multiple further dislocations [78].

**Figure 2:**
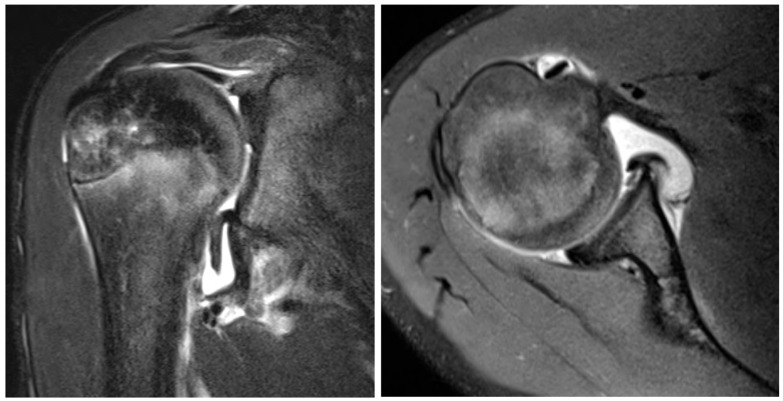
T2 weighted MRI coronal (**left**) and axial (**right**) slice of a 13-year-old boy with GLAD, SLAP, HAGL, and labral lesions.

For traumatic intra-articular pathologies such as GLAD, ALPSA, and HAGL deformities (Figure 2), the literature does not support any differences required in management between adult and paediatric populations. GLAD lesions have been reported in children as young as 6 years of age and further case reports have been reported in adolescence [23,79]. Presently, it is unknown if this lesion is more commonly seen in the paediatric/adolescent population compared to the adult population. Similarly, a systematic review showed 16% of adults were found to have an ALPSA after first-time dislocation [80], compared to 13% of adolescents aged 14 to 18 years in Nixon’s case series of adolescent rugby players who suffered a shoulder dislocation [81]. The incidence of HAGL lesions in adults range from 1% to 9%, compared to 2% in Nixon’s case series [25,80,81].

Arthroscopic Bankart repair is, therefore, a viable surgical option and can be considered early in the management of traumatic adolescent shoulder instability. Clinicians should be aware of the apparent higher risk of recurrence both with non-operative as well as operative management compared to adult cohorts.

### 5.3. Bone Loss

There is a paucity of literature in the management of bone loss in the paediatric population. However, some inferences can be made from studies of the young adult population.

#### 5.3.1. Glenoid Bone Loss

Anterior glenoid rim fractures, or bony Bankarts may be addressed with internal fixation or arthroscopic suture fixation like that recommended in young adults to prevent attritional glenoid bone loss [82]. Arthroscopic fixation is often considered in patients with a glenoid fracture as small as 9% and can also be performed in chronic cases as the blood supply is often preserved [82,83,84]. In larger glenoid rim fractures not suitable for fixation, Latarjet procedure is another option (see Section 5.4).

Ellis et al. found 48% of paediatric patients (average age 15.1 years and age range 6.5 to 18.1 years) with recurrent instability had GBL; 27% of these patients had ‘critical’ bone loss defined as 20% or greater [85]. Male, older, and taller patients, especially those who had their first-time dislocation during a sporting contest, were more likely to have glenoid bone loss [85]. Ozturk et al. found 87% of patients who underwent arthroscopic stabilisation, some of whom had GBL (<20%), were able to return to sport, and no correlation was found between GBL and the risk of recurrence [73]. This contradicts most of the literature [20,77]. In high-risk sports cohorts, a higher recurrence rate has been associated with as little as 13.5% bone loss [86]. Cheng et al. also found that adolescents (aged 15.9 years ± 1.4 years) who failed arthroscopic surgery had an average of 10% GBL compared to 5% in the success group [77].

#### 5.3.2. Hill-Sachs Lesion and Bipolar Bone Loss

A HSL can increase in size following multiple instability events [87]. A larger, off-track HSL is associated with a higher risk of recurrent instability [87]. Adolescents have been shown to have a greater likelihood of suffering an off-track HSL at a rate of 9.4 times that of adults [42]. The reason for this is uncertain but may be due to softer bone or higher impact collision sports. Higher incidences of HSLs may be a possible reason for the higher failure rate of both non-operative management and arthroscopic stabilisation in adolescents [88].

Remplissage is a viable option in augmenting arthroscopic capsulolabral repair. It involves a non-anatomic transfer of the infraspinatus tendon and posterior capsule into the Hills-Sachs defect. Hughes reported in a retrospective study that the addition of remplissage to Bankart repair in adolescents and young adults (aged 18.2 years ± 2.6 years) resulted in a reduced recurrence rate and improved outcome scores [8]. Recurrence occurred in two patients with bipolar bone loss and relatively high GBL of 16% and 18% [8]. In an older cohort including adolescents (aged 24.25 years ± 6.45 years (16–37)), Bah reported similar results in those who underwent a Latarjet procedure versus those who underwent arthroscopic Bankart repair with remplissage, despite the occurrence of HSLs < 30% and GBL < 30% [89,90]. Remplissage is associated with a reduction in shoulder external rotation (ER) of approximately eight to nine degrees in the adult population [91]. ER loss in adolescents with remplissage is unclear as the literature is sparse, however, Hughes reported ER asymmetry (10° less than on the contralateral side) in 57% of his cohort [8].

### 5.4. Latarjet

Although the Latarjet procedure has an established track record and is frequently used in the adult population, concerns exist with its routine use in children and adolescents due to the risk of osteoarthritis, the effects on the immature skeleton, and the morbidity of its complications. Most used in the setting of bone loss or failure after capsulolabral repair, it has become increasingly used as a primary procedure in high-risk patients [92].

Waltenspül reported in an adolescent population (16.4 years’ range and aged 13 to 18 years) that Latarjet resulted in lower recurrence rates than Bankart repair alone [9]. Patients underwent Latarjet (*n* = 30) if they had glenoid bone loss >15%, were a high-risk athlete, or had recurrence following a previous Bankart repair. In the Bankart group, failure occurred in 57% of patients after a mean of four years. In comparison, 6% (two patients) in the Latarjet group had treatment failure [9]. Two patients in the Latarjet group suffered coracoid process fractures, which are a risk in patients with smaller coracoids, and one patient suffered an axillary nerve palsy. The safety and success of the Latarjet in adolescence is well documented in the broader literature [93,94,95,96]. There is minimal risk of growth disturbances and patients have high rates of return to pre-injury levels of sport, with similar recovery times compared to Bankart repairs [93,94,95,96].

Risk of late degenerative changes has been a concern in the use of Latarjet, particularly in the younger population. In the adult population, there has been no definite increased risk established. In a systematic review, Latarjet is associated with a 25% risk of moderate or severe osteoarthritis at 5 years, which is not dissimilar to arthroscopic stabilisation (26%) [97,98]. However, a 4.9 times increased risk of developing OA was reported if the coracoid bone block was overhanging relative to the glenoid articular surface [98]. In another recent systematic review, Verweij et al. reported that the risk of developing OA was in fact lower with Latarjet than with arthroscopic Bankart repair or non-operative treatment [99]. This would suggest that the development of post dislocation arthropathy may be more attributable to natural history rather than surgery. However, long-term data remains lacking and given the potentially devastating and irreversible impact of early OA in adolescence, controversy remains about its routine use as a primary surgical option.

Scoring systems exist for helping to decide between Latarjet versus Bankart repair. The Instability Severity Index Score (ISIS) is commonly used and scores of over three have been shown to predict the failure of Bankart repairs [20]. However, its validity has been questioned as it may bias the Latarjet procedure [100]. The Glenoid Track Instability Management Score (GTIMS), adopted from the ISIS score, uses, in addition, 3D CT and bipolar tracking analyses to predict the failure of arthroscopic capsule-labral repairs more accurately [101]. It reduces the number of Latarjet procedures without notable differences in clinical outcomes. A limitation of the GTIMS is that validation studies have not yet compared Latarjet to Bankart with remplissage augmentation for off-track lesions, which could further reduce the need for Latarjet. No existing scoring system has been specifically validated for children and adolescents, to the best of the authors’ knowledge.

The complication rate of the Latarjet procedure has been reported to be as high as 30%, with a re-operation rate of 7% [102]. This includes graft resorption, graft fracture, malunion, fixation failure, infection, and nerve injury. Failure is most often caused by graft resorption and subsequent recurrent instability or prominent screw (12%) or by non-union (9%) of Latarjet procedures in young adults [102]. Options for failed Latarjet include an autologous graft from an iliac crest, distal clavicle, or allograft [103,104,105,106]. Distal clavicle autografts would, however, not be applicable to a paediatric population due to disruption of the growing lateral clavicle as well as its higher cartilaginous component [107].

## 6. Multidirectional Instability

**Figure 3:**
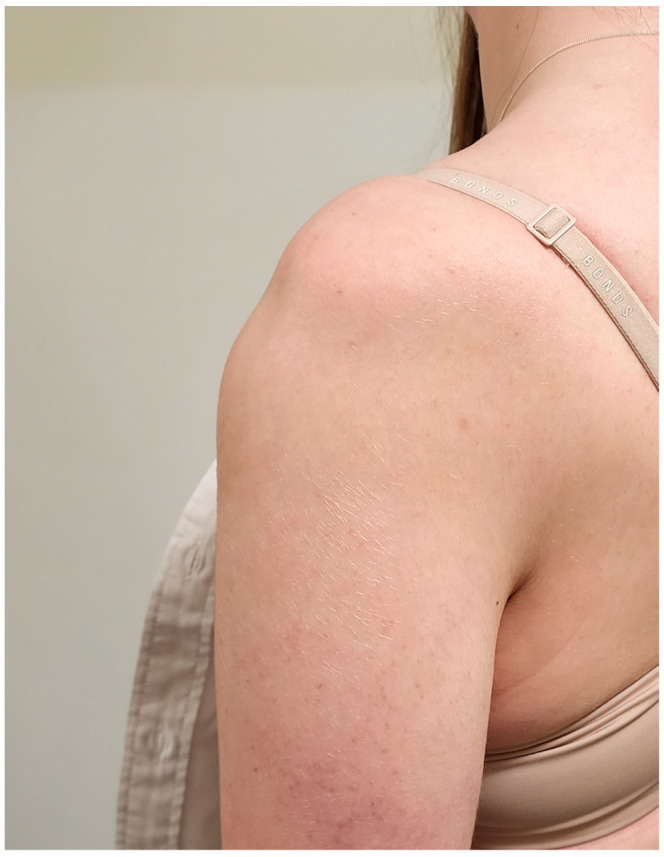
Static sulcus sign in a 14-year-old patient with MDI and static, involuntary inferior instability.

MDI is instability of the shoulder in two or more directions, with inferior being a necessary direction (Figure 3) [31]. It is seen in patients with generalised ligamentous laxity (either idiopathic or part of EDS or other syndrome), or associated with repetitive microtrauma. MDI is more common in younger patients, hence its inclusion in this review. However, there is little literature specifically addressing MDI in the paediatric population but rather studies that include both adults and adolescents.

### 6.1. Natural History

The natural history of atraumatic shoulder instability is not well documented, but it is understood that patients can get better without surgical treatment [21,108]. Contributing factors to non-operative success include an adequate response to physiotherapy as a primary treatment and gradual stiffening of soft tissue and ligaments with increasing age [21,108,109]. Moreover, older adults rarely present with MDI. A large Japanese study with a five year follow-up on the natural history of MDI patients found that patients are eight times more likely to recover from MDI if they do not play overhead sports and 9% of patients recover without physical therapy or surgical treatment [108]. The average age of onset of perception of symptomatic shoulder instability is 14 years of age. If spontaneous recovery did occur, 85.7% of women recovered before 24 years of age and 95.5% of men recovered at 20 years of age [108].

### 6.2. Physiotherapy

The mainstay of treatment for MDI without structural damage being shown on an MRI is physiotherapy. Several studies have shown initial physiotherapy leads to better patient-reported outcome measures than surgical management [21]. In MDI, there is a failure of static shoulder stabilisers, and physiotherapy programs focus on stabilising the scapula, improving neuromuscular control, proprioception, and activity modification [110]. Watson has shown the greatest success in their rehabilitation program, superseding the Burkhead and Rockwood program [39]. Watson’s program initially focuses on scapula stabilizers, then the rotator cuff, then sport or work-specific programs. This program leads to improvements in the Melbourne Instability Shoulder Score (MISS), Oxford Instability Shoulder Score (OISS), strength, and function in as little as 12 weeks [12,111]. It is expected that patients continue to exercise three to four times per week following the conclusion of the program. Risk factors that predict poor response to physiotherapy are difficulties with daily routines, higher degrees of laxity, and unilateral involvement [109].

#### Adjuncts to Physiotherapy

Strapping can help patients by assisting with proprioception. It allows patients to feel supported by altering the posture and position of the scapula and shoulder. Evidence is however limited, and the early literature revealed neoprene body garments can help with joint re-position sense (JRS) but kinesio-tape was shown to be detrimental [112,113]. A shoulder orthosis developed by Ide et al. can augment the results of physiotherapy. It aims to increase scapula inclination and straighten the thoracic spine and has shown an 85% improvement in the modified Rowe score when used in conjunction with the Burkhead and Rockwood rehabilitation program, compared to 75% in the control group [114].

Electromyography can assist physiotherapy regimes to assist with real-time biofeedback. It encourages patients to isolate relevant muscle groups for targeted rehabilitation [3,115]. Functional electrical stimulation has shown benefit in patients with shoulder subluxation post stroke or spinal injury [116]. It is in its early stages of use for atraumatic instability but may be more beneficial in patients who have a muscle patterning element (See Section 7.2.2).

### 6.3. Surgical Options

When non-operative management and physiotherapy have been exhausted, surgical stabilisation for MDI can be considered. Prior to surgery, some authors recommend psychological assessment, as concomitant psychological comorbidities are risk factors for the failure of both conservative and surgical management [117]. The aim of surgical intervention is to augment the static stabilisers in the direction of the patient’s instability and to reduce the capsular volume.

#### 6.3.1. Capsular Plication

While open capsular shift is an option and has been shown to have reasonable results in a cohort of adolescents with EDS and shoulder MDI [118], arthroscopic capsular plication is the more popular option for most surgeons as it confers the advantage of allowing visualisation and plication of the capsule in all shoulder quadrants. In a recent review of 42 adolescent shoulders with MDI, which was defined by a positive drive through sign, there was a sulcus sign and/or posterior or anterior draw. The study found success in improved clinical outcomes as measured using the Single Assessment Numerical Evaluation (SANE) score [13]. There was a 26% recurrence of instability and a time to re-operation of 1.9 years post-op in the failure group. Return to sport was achieved in 56% of patients. Aside from a lower rate of return to sport, outcomes were like those for Bankart repairs for traumatic unidirectional instability. The authors found no association between failure of surgery and younger age, female sex, or generalised ligamentous laxity, which are known risk factors in the adult population. Of note, in this series, only five of the studied cohort had MDI associated with generalised ligament laxity and the majority (70%) of patients had a Bankart lesion, suggesting that they were likely suffering from a combination of MDI and traumatic instability rather than true Polar II/atraumatic instability [13]. Greiwe reported excellent results in a case series of ten adolescents with shoulder MDI. After an average follow-up time of 31 months, there were no cases of recurrent instability and ASES scores improved dramatically from 52.2 ± 18.7 to 85.9 ± 14.9 [14]. For adults, the literature also shows promising results, with rate of recurrent instability following arthroscopic plication ranging from 8 to 31%, and a rate of return to sport of 50 to 86% [119]. Witney-Lagen also had success in their study of mostly adults with MDI, showing a 94% satisfaction rate. There was no significant difference between patients below and over 25 years of age, but those with higher Beighton scores had less marked improvement [120].

#### 6.3.2. Inferior Glenohumeral Ligament Reconstruction

Case reports are available describing the use of tendon graft to reconstruct the IGHL arthroscopically [121,122,123,124]. Techniques vary, but the general intention is to reconstruct both the anterior and posterior bands of the IGHL to recreate the deficient inferior sling of the glenohumeral joint. IGHL reconstruction shows promising results in those with severe MDI and ligamentous laxity who have failed other forms of surgical intervention [121,122,123,124]. More research is needed to confirm the safety and long-term efficacy of this technique.

## 7. Muscle Patterning Instability

Muscle patterning instability is a rare form of shoulder instability that occurs as a result of aberrant muscle contractions and disorganization of the muscle patterning at the glenohumeral and scapholothoracic articulation. The shoulder may dislocate due to the head of the humerus being pulled out of the glenoid socket due to over and underactivity of the stabilizing musculature [15,34].

### 7.1. Classification

Moroder et al., in a recent study, proposed using the term functional shoulder instability (FSI) to describe atraumatic shoulder instability without ligamentous laxity [15]. By analysing in detail a cohort of patients with FSI, the authors classified FSI into four subgroups. These were predominantly based on pathomechanism and volitional control. They were grouped as positional and non-positional, controllable, and non-controllable. Positional FSI refers to instability at a certain point during the motion of the arm; this can be either controllable or non-controllable. Non-positional refers to spasmodic ‘tic’-like contractions with the arm in a near neutral position leading to instability events and this can also either be controllable or not [15]. Positional and non-controllable instability was the most represented in the study (72%). Patients with controllable instability are likely under-represented as they often do not perceive their pathology as an impairment but rather an enhanced ability. Non-positional and non-controllable FSI patients suffer the greatest morbidity. Patients are then further divided into the direction of their instability. Unidirectional and posterior were found to be the most common (78%), triggered by the shoulder in a flexed and internally rotated position [15]. Most of the patients assessed had no structural abnormalities in their shoulders, although a small portion of patients had mild glenoid dysplasia (24%) and labral damage (16%). A total of 89% of patients did have scapular dyskinesia but it was impossible to discern if this was a cause or consequence of FSI [15].

### 7.2. Management

Current evidence shows instability due to abnormal muscle patterning is best managed non-operatively for all its forms. It is highly unlikely for permanent structural damage to occur from a muscle patterning dislocation due to the low concavity compression forces. This allows the clinician to enact a watch and wait approach for each patient more comfortably. Psychological comorbidities, whether primary or secondary, can further complicate the management of these patients [15].

#### 7.2.1. Psychology

It is important to consider the possibility of underlying psychological contributors in the management of paediatric atraumatic shoulder instability. Neurophysiological dysregulation in children such as that described in functional neurological disorders (FND) is not uncommon. In children affected by FND, there is a dysregulation of the stress system and overflooding of the neural networks, which, in turn, disrupt the motor and sensory processing centre. These symptoms can occur without consciousness or intention and disrupt function in many other aspects of the child’s daily life. An underlying stressor may or may not be identified. On the other hand, psychological disorders can also give rise to secondary chronic morbidity associated with recalcitrant shoulder instability. Therefore, care must be taken in deftly assessing this paediatric population. When psychosocial comorbidities and neurophysiological dysregulation is identified, a biopsychosocial evaluation and an interdisciplinary approach is required. Therefore, it is important for the surgeon and physiotherapist to optimise non-surgical management such as goal directed rehabilitation, focus on return to function, and include referrals to mental health specialists and general paediatricians for co-management.

#### 7.2.2. Voluntary

Voluntary dislocators are not uncommon among children. Those without associated psychological disorders or complaints do not need any treatment. They often present to shoulder specialists due to concern from their parents. Parents often report that their children can perform “unnatural” party tricks with their shoulders. The voluntary dislocator with concomitant mental health illness, who may be using dislocation events for secondary gain, is well known to clinicians and is a very difficult problem to address [115]. For these patients, it is important that they are seen by a specialist physiotherapist, and that the physiotherapist does their best to earn the patient’s trust, ideally with the parents not present, if appropriate. If psychosocial risk factors are present, they should be referred to a psychiatrist. If a clear psychosocial trigger for their instability events can be identified, this may help with developing coping strategies. Furthermore, as these patients often present to emergency departments with ever-changing receiving clinicians, a multi-disciplinary management plan should be made in conjunction with orthopaedics, physiotherapy, psychiatry, and emergency staff in order to prevent admission, minimise treatment time, and prevent unnecessary investigation and procedures [125].

#### 7.2.3. Non-Voluntary

These patients can have truly painful and difficult to manage pathologies and care should be taken not to mistake them for care seeking. In these patients, mental health conditions are often secondary to the trauma caused by the instability and sometimes neglect from health professionals during their adolescence. ED management plans should also be developed for these patients to make their hospital experience less painful and prevent somatization.

As the results of surgery can be unpredictable, and the pathology is often self-limiting and improves with age, a watch and wait policy is a reasonable approach [126]. However, this is not satisfactory for those who suffer a great deal. Physiotherapy should focus on core stabilisation, coordination, strengthening, and biofeedback [3,127]. Studies that report success with physiotherapy report requiring three to four in-person specialist sessions per week, which is not feasible for many population groups [3,127]. EMG can sometimes be helpful in identifying the over- or underactive muscle. A botulinum toxin injection can also be introduced to the identified overactive muscle under ultrasound guidance. Systemic muscle relaxants have been trialled but are generally avoided as it is not possible to target the isolated overactive muscle groups and can lead to side effects and dependencies.

In recent years, there has been a promising advancement with the use of a ‘shoulder pacemaker’ [16]. Moroder assessed the use of a targeted electric muscle stimulation device in three patients with posterior positional FSI. Electrodes were placed in the rhomboids, trapezius, infraspinatus, and posterior deltoid and applied a previously determined level of electric stimulation to activate the necessary muscles. When worn, each patient was able to move their shoulder pain-free without dislocating. These promising results may one day allow use of these devices in conjunction with physiotherapy to help re-establish muscular balance [16].

## 8. Clinical Relevance

The ability to see paediatric and adolescent shoulder instability as its own entity with its own unique diagnostic and management strategies separate from adult ones will enable clinicians and patients to make better treatment decisions going forward. (See Table 2) With a stronger understanding of the different types of instability and their forms of management, dislocation recurrence will reduce, patient outcomes should improve, hospital emergency departments will be less strained, there will be a decrease in the use of unnecessary diagnostic imaging, and the incidence of glenohumeral osteoarthritis will reduce. In addition, by breaking down paediatric and adolescent shoulder instability into its three main subtypes and highlighting the challenge of interpreting the literature with inconsistent definitions and crossover of instability aetiology, we would hope that further research can accurately assess subtypes and combination forms of instability. This would allow clinicians to be better equipped with more reliable and evidence-based management strategies going forward.

### 8.1. Strengths

The strength of this review is that it is an in-depth analysis that encompasses most forms of paediatric and adolescent shoulder instability from pathology to presentation to management. The literature presented includes important older papers with a focus on the current literature available. There are efforts to clearly separate the three predominant forms of shoulder instability as separate entities with different management strategies that overlap.

### 8.2. Limitations

As a narrative review, there is no quantitative synthesis of data such as a meta-analysis. Without a structured methodology for searching, selecting, and evaluating studies as is seen in systematic reviews, there is an inherent bias and risk of over generalization when selecting and interpreting the literature.

## 9. Conclusions

Paediatric and adolescent shoulder instability is a complex and clinically challenging area. It straddles a grey zone between several types of shoulder instability. In the younger child, shoulder instability without structural abnormalities predominates. Whereas in the older adolescent, the pathology is more likely to reflect that seen in adults, with structural injuries such as HSLs and Bankart lesions. Ligamentous laxity and muscle patterning abnormalities are more likely to exist in the younger patient, adding to the diagnostic challenges. For traumatic instability with structural lesions, adolescents are at a very high risk of poor outcomes with non-operative management, and stabilisation should be considered early with consideration given to adjuncts such as remplissage based on the characteristics of bone loss. The Latarjet is a viable option for adolescents and can be used in cases of failed arthroscopic treatment or for high-risk patients, but the side-effect profile should be carefully considered. For MDI, the mainstay of treatment remains specialist long-term and regular physiotherapy with arthroscopic plication as the preferred option if conservative means fail. Finally, for shoulder instability arising from predominant muscle patterning problems, the focus should be on gaining neuromuscular control, with the promise of newer technology such as the shoulder pacemaker to augment rehabilitation programs.

## Figures and Tables

**Table 1:** Epidemiological studies on incidence of shoulder instability and recurrence.

Author	Population	Time Period	Location	Age Group	Rate of Primary Dislocation (Per 100,000 Person-Years)	Rate of Repeat Dislocation	Time to Repeat Dislocation
Leroux et al. [5]	Patients 10 to 16 who had a primary anterior dislocation requiring reduction.	2002 to 2010	Toronto, Canada	10	1.85	0%	Median time to repeat dislocation 0.8 years. 99% of repeat dislocation occurred within 5.3 years. Minimum of 2 year follow-up
				11	2.05	15%	
				12	4.38	22%	
				13	9.61	25%	
				14	17.4	37.2%	
				15	32.72	40.8%	
				16	96.95	42.3%	
Yapp et al. [35]	All patients 14 and under who sustained a shoulder dislocation requiring reduction.	2008 to 2019	Ontario, Canada	0 to 14 yoa	2.5	43% (*n* = 41) Higher in those with closed physis (12/17) as opposed to open: (6/24)	Median of 14.7 months
Longo et al. [37]	All patients 14 and under who were hospitalised due to shoulder dislocation	2001 to 2014	Italy	0 to 14	0.3	NRC	N/A
Zacchilli et al. [36]	All emergency department shoulder dislocation presentations	2002 to 2006	USA	0 to 9	0.92	NRC	N/A
				10 to 19	39.71	NRC	

NRC: Not recorded. N/A: Not applicable.

**Table 2:** Summary of possible differences in paediatric and adolescent patients compared to adult patients.

Subject	Difference
Stanmore types	More polar type II/III and combined types than seen in adults
Ligamentous laxity	More of a contributor than in adults
Presence of GLAD/ALPSA/HAGL	No evidence of difference in incidence compared to adults
Glenoid bone loss	May be less predictive of failure with Bankart repair surgery than in adults
Hill-Sachs lesions	Greater likelihood of off-track Hill-Sachs lesions than in adults
Non-operative management	More likely to fail if structural injuries are present on MRI. If structural injuries are present, the risk of failure increases with decreasing age.
Arthroscopic Bankart repair	Higher risk of post-operative recurrence than adults
Bankart + remplissage	Should perhaps be utilized more due to higher risk of post-operative recurrence
Latarjet procedure	Equally effective vs. adults. However with smaller coracoids, the risk of coracoid fracture is higher and the risk of late osteoarthritis if the graft is overhanging can be devastating in younger patients
Multidirectional instability	More common than in adults
Capsulorraphy for MDI	Similar role in younger age group vs. adults. Reserved as a last resort.
Functional shoulder instability (FSI)	Possibly more common than in adults.

## References

[1] Goldberg A.S., Moroz L., Smith A., Ganley T. (2007). Injury surveillance in young athletes. Sports Med..

[2] Nicolozakes C.P., Li X., Uhl T.L., Marra G., Jain N.B., Perreault E.J., Seitz A.L. (2021). Interprofessional Inconsistencies in the Diagnosis of Shoulder Instability: Survey Results of Physicians and Rehabilitation Providers. Int. J. Sports Phys. Ther..

[3] Jaggi A., Lambert S. (2010). Rehabilitation for shoulder instability. Br. J. Sports Med..

[4] Hill B., Khodaee M. (2022). Glenohumeral Joint Dislocation Classification: Literature Review and Suggestion for a New Subtype. Curr. Sports Med. Rep..

[5] Leroux T., Ogilvie-Harris D., Veillette C., Chahal J., Dwyer T., Khoshbin A., Henry P., Mahomed N., Wasserstein D. (2015). The epidemiology of primary anterior shoulder dislocations in patients aged 10 to 16 years. Am. J. Sports Med..

[6] Leland D.P., Bernard C.D., Keyt L.K., Krych A.J., Dahm D.L., Sanchez-Sotelo J., Camp C.L. (2020). An age-based approach to anterior shoulder instability in patients under 40 years old: Analysis of a US population. Am. J. Sports Med..

[7] Gigis I., Heikenfeld R., Kapinas A., Listringhaus R., Godolias G. (2014). Arthroscopic versus conservative treatment of first anterior dislocation of the shoulder in adolescents. J. Pediatr. Orthop..

[8] Hughes J.L., Bastrom T., Pennock A.T., Edmonds E.W. (2018). Arthroscopic Bankart repairs with and without remplissage in recurrent adolescent anterior shoulder instability with Hill-Sachs deformity. Orthop. J. Sports Med..

[9] Waltenspül M., Ernstbrunner L., Ackermann J., Thiel K., Galvin J.W., Wieser K. (2022). Long-term results and failure analysis of the open latarjet procedure and arthroscopic Bankart repair in adolescents. JBJS.

[10] Postacchini F., Gumina S., Cinotti G. (2000). Anterior shoulder dislocation in adolescents. J. Shoulder Elb. Surg..

[11] Cordischi K., Li X., Busconi B. (2009). Intermediate outcomes after primary traumatic anterior shoulder dislocation in skeletally immature patients aged 10 to 13 years. Orthopedics.

[12] Warby S.A., Ford J.J., Hahne A.J., Watson L., Balster S., Lenssen R., Pizzari T. (2018). Comparison of 2 exercise rehabilitation programs for multidirectional instability of the glenohumeral joint: A randomized controlled trial. Am. J. Sports Med..

[13] Mitchell B.C., Siow M.Y., Carroll A.N., Pennock A.T., Edmonds E.W. (2021). Clinical Outcomes, Survivorship, and Return to Sport After Arthroscopic Capsular Repair With Suture Anchors for Adolescent Multidirectional Shoulder Instability: Results at 6-Year Follow-up. Orthop. J. Sports Med..

[14] Greiwe R.M., Galano G., Grantham J., Ahmad C.S. (2012). Arthroscopic stabilization for voluntary shoulder instability. J. Pediatr. Orthop..

[15] Moroder P., Danzinger V., Maziak N., Plachel F., Pauly S., Scheibel M., Minkus M. (2020). Characteristics of functional shoulder instability. J. Shoulder Elb. Surg..

[16] Moroder P., Minkus M., Böhm E., Danzinger V., Gerhardt C., Scheibel M. (2017). Use of shoulder pacemaker for treatment of functional shoulder instability. Obere Extrem..

[17] Lin K.M., James E.W., Spitzer E., Fabricant P.D. (2018). Pediatric and adolescent anterior shoulder instability: Clinical management of first-time dislocators. Curr. Opin. Pediatr..

[18] Olds M., Donaldson K., Ellis R., Kersten P. (2016). In children 18 years and under, what promotes recurrent shoulder instability after traumatic anterior shoulder dislocation? A systematic review and meta-analysis of risk factors. Br. J. Sports Med..

[19] Shanmugaraj A., Chai D., Sarraj M., Gohal C., Horner N.S., Simunovic N., Athwal G.S., Ayeni O.R. (2021). Surgical stabilization of pediatric anterior shoulder instability yields high recurrence rates: A systematic review. Knee Surg. Sports Traumatol. Arthrosc..

[20] Verweij L.P.E., van Spanning S.H., Grillo A., Kerkhoffs G.M.M.J., Priester-Vink S., van Deurzen D.F.P., van den Bekerom M.P.J. (2021). Age, participation in competitive sports, bony lesions, ALPSA lesions,  > 1 preoperative dislocations, surgical delay and ISIS score > 3 are risk factors for recurrence following arthroscopic Bankart repair: A systematic review and meta-analysis of 4584 shoulders. Knee Surg. Sports Traumatol. Arthrosc..

[21] Warby S.A., Pizzari T., Ford J.J., Hahne A.J., Watson L. (2016). Exercise-based management versus surgery for multidirectional instability of the glenohumeral joint: A systematic review. Br. J. Sports Med..

[22] Bankart A.B. (1923). Recurrent or habitual dislocation of the shoulder-joint. Br. Med. J..

[23] Neviaser T.J. (1993). The GLAD lesion: Another cause of anterior shoulder pain. Arthrosc. J. Arthrosc. Relat. Surg..

[24] Neviaser T.J. (1993). The anterior labroligamentous periosteal sleeve avulsion lesion: A cause of anterior instability of the shoulder. Arthrosc. J. Arthrosc. Relat. Surg..

[25] Wolf E.M., Cheng J.C., Dickson K. (1995). Humeral avulsion of glenohumeral ligaments as a cause of anterior shoulder instability. Arthrosc. J. Arthrosc. Relat. Surg..

[26] Provencher M.T., Midtgaard K.S., Owens B.D., Tokish J.M. (2021). Diagnosis and management of traumatic anterior shoulder instability. JAAOS-J. Am. Acad. Orthop. Surg..

[27] Hill H.A., Sachs M.D. (1940). The Grooved Defect of the Humeral Head. Radiology.

[28] Kim S.-H., Ha K.-I., Yoo J.-C., Noh K.-C. (2004). Kim’s lesion: An incomplete and concealed avulsion of the posteroinferior labrum in posterior or multidirectional posteroinferior instability of the shoulder. Arthroscopy.

[29] Castagna A., Garofalo R., Conti M., Randelli M. (2005). Reverse HAGL: A possible complication of a tight anterior gleno-humeral stabilization. Chir. Degli Organi Mov..

[30] Cicak N. (2004). Posterior dislocation of the shoulder. J. Bone Jt. Surgery. Br. Vol..

[31] Longo U.G., Rizzello G., Loppini M., Locher J., Buchmann S., Maffulli N., Denaro V. (2015). Multidirectional instability of the shoulder: A systematic review. Arthrosc. J. Arthrosc. Relat. Surg..

[32] Rong Y.-H., Zhang G.-A., Wang C., Ning F.-G. (2008). Quantification of type I and III collagen content in normal human skin in different age groups. Chin. J. Burn..

[33] Rodeo S.A., Suzuki K., Yamauchi M., Bhargava M., Warren R.F. (1998). Analysis of Collagen and Elastic Fibers in Shoulder Capsule in Patients with Shoulder Instability. Am. J. Sports Med..

[34] Jaggi A., Noorani A., Malone A., Cowan J., Lambert S., Bayley I. (2012). Muscle activation patterns in patients with recurrent shoulder instability. Int. J. Shoulder Surg..

[35] Yapp L.Z., Baxendale-Smith L., Nicholson J.A., Gaston M.S., Robinson C.M. (2021). Traumatic glenohumeral dislocation in pediatric patients is associated with a high risk of recurrent instability. J. Pediatr. Orthop..

[36] Zacchilli M.A., Owens B.D. (2010). Epidemiology of shoulder dislocations presenting to emergency departments in the United States. JBJS.

[37] Longo U.G., Salvatore G., Locher J., Ruzzini L., Candela V., Berton A., Stelitano G., Schena E., Denaro V. (2020). Epidemiology of Paediatric Shoulder Dislocation: A Nationwide Study in Italy from 2001 to 2014. Int. J. Environ. Res. Public Health.

[38] Hovelius L., Olofsson A., Sandström B., Augustini B.-G., Krantz L., Fredin H., Tillander B., Skoglund U., Salomonsson B., Nowak J. (2008). Nonoperative treatment of primary anterior shoulder dislocation in patients forty years of age and younger: A prospective twenty-five-year follow-up. JBJS.

[39] Watson L., Warby S., Balster S., Lenssen R., Pizzari T. (2016). The treatment of multidirectional instability of the shoulder with a rehabilitation program: Part 1. Shoulder Elb..

[40] Hawkins R.J., Schutte J.P., Janda D.H., Huckell G.H. (1996). Translation of the glenohumeral joint with the patient under anesthesia. J. Shoulder Elb. Surg..

[41] Rowe C.R., Zarins B. (1981). Recurrent transient subluxation of the shoulder. J. Bone Jt. Surg. Am..

[42] Jobe F.W., Kvitne R.S., Giangarra C.E. (1989). Shoulder pain in the overhand or throwing athlete. The relationship of anterior instability and rotator cuff impingement. Orthop. Rev..

[43] Warth R.J., Millett P.J. (2015). Physical Examination of the Shoulder.

[44] Krishnan S.G., Hawkins R.J., Warren R.F. (2004). The Shoulder and the Overhead Athlete.

[45] Gagey O. (2001). The hyperabduction test: An assessment of the laxity of the inferior glenohumeral ligament. J. Bone Jt. Surg. Br. Vol..

[46] Beighton P., Solomon L., Soskolne C. (1973). Articular mobility in an African population. Ann. Rheum. Dis..

[47] Jankauskas L., Rüdiger H.A., Pfirrmann C.W., Jost B., Gerber C. (2010). Loss of the sclerotic line of the glenoid on anteroposterior radiographs of the shoulder: A diagnostic sign for an osseous defect of the anterior glenoid rim. J. Shoulder Elb. Surg..

[48] Meyer D.C., Ernstbrunner L., Boyce G., Imam M.A., El Nashar R., Gerber C. (2019). Posterior acromial morphology is significantly associated with posterior shoulder instability. JBJS.

[49] Beeler S., Leoty L., Hochreiter B., Carrillo F., Götschi T., Fischer T., Fürnstahl P., Gerber C. (2021). Similar scapular morphology in patients with dynamic and static posterior shoulder instability. JSES Int..

[50] Huijsmans P.E., Haen P.S., Kidd M., Dhert W.J., van der Hulst V.P., Willems W.J. (2007). Quantification of a glenoid defect with three-dimensional computed tomography and magnetic resonance imaging: A cadaveric study. J. Shoulder Elb. Surg..

[51] Gyftopoulos S., Hasan S., Bencardino J., Mayo J., Nayyar S., Babb J., Jazrawi L. (2012). Diagnostic accuracy of MRI in the measurement of glenoid bone loss. Am. J. Roentgenol..

[52] Gyftopoulos S., Beltran L.S., Bookman J., Rokito A. (2015). MRI Evaluation of Bipolar Bone Loss Using the On-Track Off-Track Method: A Feasibility Study. Am. J. Roentgenol..

[53] Yellin J.L., Fabricant P.D., Anari J.B., Neuwirth A.L., Ganley T.J., Chauvin N.A., Lawrence J.T. (2021). Increased Glenoid Index as a Risk Factor for Pediatric and Adolescent Anterior Glenohumeral Dislocation: An MRI-Based, Case-Control Study. Orthop. J. Sports Med..

[54] Weber A.E., Bolia I.K., Horn A., Villacis D., Omid R., Tibone J.E., White E., Hatch G.F. (2021). Glenoid Bone Loss in Shoulder Instability: Superiority of Three-Dimensional Computed Tomography over Two-Dimensional Magnetic Resonance Imaging Using Established Methodology. Clin. Orthop. Surg..

[55] Stefaniak J., Kubicka A.M., Wawrzyniak A., Romanowski L., Lubiatowski P. (2020). Reliability of humeral head measurements performed using two- and three-dimensional computed tomography in patients with shoulder instability. Int. Orthop..

[56] Park K.-J., Jeong H.-S., Park J.-K., Cha J.-K., Kang S.-W. (2019). Evaluation of Inferior Capsular Laxity in Patients with Atraumatic Multidirectional Shoulder Instability with Magnetic Resonance Arthrography. KJR.

[57] Lim C.-O., Park K.-J., Cho B.-K., Kim Y.-M., Chun K.-A. (2016). A new screening method for multidirectional shoulder instability on magnetic resonance arthrography: Labro-capsular distance. Skelet. Radiol..

[58] Lee H.J., Kim N.R., Moon S.G., Ko S.M., Park J.-Y. (2013). Multidirectional instability of the shoulder: Rotator interval dimension and capsular laxity evaluation using MR arthrography. Skelet. Radiol..

[59] Lephart S.M., Warner J.J., Borsa P.A., Fu F.H. (1994). Proprioception of the shoulder joint in healthy, unstable, and surgically repaired shoulders. J Shoulder Elb. Surg.

[60] Fyhr C., Gustavsson L., Wassinger C., Sole G. (2015). The effects of shoulder injury on kinaesthesia: A systematic review and meta-analysis. Man. Ther..

[61] Lubiatowski P., Ogrodowicz P., Wojtaszek M., Romanowski L. (2019). Bilateral shoulder proprioception deficit in unilateral anterior shoulder instability. J. Shoulder Elb. Surg..

[62] Ager A., Roy J.-S., Roos M., Fournier Belley A., Cools A., Hébert L. (2017). Shoulder proprioception: How is it measured and is it reliable? A systematic review. J. Hand Ther..

[63] Ogawa K., Yoshida A., Ikegami H. (2006). Osteoarthritis in shoulders with traumatic anterior instability: Preoperative survey using radiography and computed tomography. J. Shoulder Elb. Surg..

[64] Brophy R.H., Marx R.G. (2005). Osteoarthritis following shoulder instability. Clin. Sports Med..

[65] Whelan D.B., Litchfield R., Wambolt E., Dainty K.N., Joint Orthopaedic Initiative for National Trials of the Shoulder (JOINTS) (2014). External rotation immobilization for primary shoulder dislocation: A randomized controlled trial. Clin. Orthop. Relat. Res..

[66] Paterson W.H., Throckmorton T.W., Koester M., Azar F.M., Kuhn J.E. (2010). Position and duration of immobilization after primary anterior shoulder dislocation: A systematic review and meta-analysis of the literature. JBJS.

[67] Watson S., Allen B., Grant J.A. (2016). A clinical review of return-to-play considerations after anterior shoulder dislocation. Sports Health.

[68] Lampert C., Baumgartner G., Slongo T., Kohler G., Horst M. (2003). Traumatic shoulder dislocation in children and adolescents. Eur. J. Trauma.

[69] Deitch J., Mehlman C.T., Foad S.L., Obbehat A., Mallory M. (2003). Traumatic anterior shoulder dislocation in adolescents. Am. J. Sports Med..

[70] Kawasaki T., Ota C., Urayama S., Maki N., Nagayama M., Kaketa T., Takazawa Y., Kaneko K. (2014). Incidence of and risk factors for traumatic anterior shoulder dislocation: An epidemiologic study in high-school rugby players. J. Shoulder Elb. Surg..

[71] Kwapisz A., Shanley E., Momaya A.M., Young C., Kissenberth M.J., Tolan S.J., Lonergan K.T., Wyland D.J., Hawkins R.J., Pill S.G. (2021). Does Functional Bracing of the Unstable Shoulder Improve Return to Play in Scholastic Athletes? Returning the Unstable Shoulder to Play. Sports Health.

[72] Tokish J.M., Thigpen C.A., Kissenberth M.J., Tolan S.J., Lonergan K.T., Tokish J.M., Dickens J.F., Hawkins R.J., Shanley E. (2020). The Nonoperative Instability Severity Index Score (NISIS): A simple tool to guide operative versus nonoperative treatment of the unstable shoulder. Sports Health.

[73] Ozturk B.Y., Maak T.G., Fabricant P., Altchek D.W., Williams R.J., Warren R.F., Cordasco F.A., Allen A.A. (2013). Return to sports after arthroscopic anterior stabilization in patients aged younger than 25 years. Arthrosc. J. Arthrosc. Relat. Surg..

[74] Hickey I.P., Davey M.S., Hurley E.T., Gaafar M., Delaney R.A., Mullett H. (2022). Return to play following open Bankart repair in collision athletes aged 18 years or less. J. Shoulder Elb. Surg..

[75] Shymon S.J., Roocroft J., Edmonds E.W. (2015). Traumatic anterior instability of the pediatric shoulder: A comparison of arthroscopic and open Bankart repairs. J. Pediatr. Orthop..

[76] Torrance E., Clarke C.J., Monga P., Funk L., Walton M.J. (2018). Recurrence after Arthroscopic Labral Repair for Traumatic Anterior Instability in Adolescent Rugby and Contact Athletes. Am. J. Sports Med..

[77] Cheng T.T., Edmonds E.W., Bastrom T.P., Pennock A.T. (2021). Glenoid pathology, skeletal immaturity, and multiple preoperative instability events are risk factors for recurrent anterior shoulder instability after arthroscopic stabilization in adolescent athletes. Arthrosc. J. Arthrosc. Relat. Surg..

[78] Hung N.J., Darevsky D.M., Pandya N.K. (2020). Pediatric and adolescent shoulder instability: Does insurance status predict delays in care, outcomes, and complication rate?. Orthop. J. Sports Med..

[79] Morsy M.G., Waly A.H.T., Galal M.A., Gawish H.M. (2021). Glenoid labrum articular disruption in a six-year-old child: A case report. Trauma Case Rep..

[80] Rutgers C., Verweij L.P.E., Priester-Vink S., van Deurzen D.F.P., Maas M., van den Bekerom M.P.J. (2022). Recurrence in traumatic anterior shoulder dislocations increases the prevalence of Hill-Sachs and Bankart lesions: A systematic review and meta-analysis. Knee Surg. Sports Traumatol. Arthrosc..

[81] Nixon M.F., Keenan O., Funk L. (2015). High recurrence of instability in adolescents playing contact sports after arthroscopic shoulder stabilization. J. Pediatr. Orthop. B.

[82] Godin J.A., Altintas B., Horan M.P., Hussain Z.B., Pogorzelski J., Fritz E.M., Millett P.J. (2019). Midterm results of the bony Bankart bridge technique for the treatment of bony Bankart lesions. Am. J. Sports Med..

[83] Kim Y.-K., Cho S.-H., Son W.-S., Moon S.-H. (2014). Arthroscopic repair of small and medium-sized bony Bankart lesions. Am. J. Sports Med..

[84] Sugaya H., Moriishi J., Kanisawa I., Tsuchiya A. (2005). Arthroscopic osseous Bankart repair for chronic recurrent traumatic anterior glenohumeral instability. JBJS.

[85] Ellis H.B., Seiter M., Wise K., Wilson P. (2017). Glenoid bone loss in traumatic glenohumeral instability in the adolescent population. J. Pediatr. Orthop..

[86] Shaha J.S., Cook J.B., Song D.J., Rowles D.J., Bottoni C.R., Shaha S.H., Tokish J.M. (2015). Redefining “critical” bone loss in shoulder instability: Functional outcomes worsen with “subcritical” bone loss. Am. J. Sports Med..

[87] Kinsella S.D., Chauvin N.A., Diaz T., Morey J.M., Wells L. (2015). Traumatic shoulder dislocation among adolescents: Hill-Sachs lesion volume and recurrent instability. J. Pediatr. Orthop..

[88] Lau B.C., Conway D., Curran P.F., Feeley B.T., Pandya N.K. (2017). Bipolar bone loss in patients with anterior shoulder dislocation: A comparison of adolescents versus adult patients. Arthrosc. J. Arthrosc. Relat. Surg..

[89] Bah A., Lateur G., Kouevidjin B., Bassinga J., Issa M., Jaafar A., Beaudouin E. (2018). Chronic anterior shoulder instability with significant Hill–Sachs lesion: Arthroscopic Bankart with remplissage versus open Latarjet procedure. Orthop. Traumatol. Surg. Res..

[90] Callegari J.J., McGarry M., Crook L., Adamson N.A., Fraipont G.M., Provencher M., Lee T.Q., Denard P.J. (2022). The Addition of Remplissage to Free Bone Block Restores Translation and Stiffness Compared to Bone Block alone or Latarjet in a Bipolar Bone Loss Model. Arthrosc. J. Arthrosc. Relat. Surg..

[91] Boileau P., O’Shea K., Vargas P., Pinedo M., Old J., Zumstein M. (2012). Anatomical and functional results after arthroscopic Hill-Sachs remplissage. JBJS.

[92] Min K., Fedorka C., Solberg M.J., Shaha S.H., Higgins L.D. (2018). The cost-effectiveness of the arthroscopic Bankart versus open Latarjet in the treatment of primary shoulder instability. J. Shoulder Elb. Surg..

[93] Khan A., Samba A., Pereira B., Canavese F. (2014). Anterior dislocation of the shoulder in skeletally immature patients: Comparison between non-operative treatment versus open Latarjet’s procedure. Bone Jt. J..

[94] Domos P., Chelli M., Lunini E., Ascione F., Bercik M.J., Neyton L., Godeneche A., Walch G. (2020). Clinical and radiographic outcomes of the open Latarjet procedure in skeletally immature patients. J. Shoulder Elb. Surg..

[95] Heyworth B.E., Wu M., Kramer D.E., Bae D.S. (2018). The Latarjet Procedure for Anterior Shoulder Instability in Pediatric and Adolescent Athletes. Orthop. J. Sports Med..

[96] Rossi L.A., Tanoira I., Bruchmann M.G., Pasqualini I., Ranalletta M. (2022). The Latarjet procedure in competitive athletes younger than 20 years old with a significant glenoid bone loss. Shoulder Elb..

[97] Harris J.D., Gupta A.K., Mall N.A., Abrams G.D., McCormick F.M., Cole B.J., Bach B.R., Romeo A.A., Verma N.N. (2013). Long-term outcomes after Bankart shoulder stabilization. Arthrosc. J. Arthrosc. Relat. Surg..

[98] Menon A., Fossati C., Magnani M., Boveri S., Compagnoni R., Randelli P.S. (2021). Low grade of osteoarthritis development after Latarjet procedure with a minimum 5 years of follow-up: A systematic review and pooled analysis. Knee Surg. Sports Traumatol. Arthrosc..

[99] Verweij L.P., Pruijssen E.C., Kerkhoffs G.M., Blankevoort L., Sierevelt I.N., van Deurzen D.F., van den Bekerom M.P. (2021). Treatment type may influence degree of post-dislocation shoulder osteoarthritis: A systematic review and meta-analysis. Knee Surg. Sports Traumatol. Arthrosc..

[100] Bouliane M., Saliken D., Beaupre L., Silveira A., Saraswat M., Sheps D. (2014). Evaluation of the Instability Severity Index Score and the Western Ontario Shoulder Instability Index as predictors of failure following arthroscopic Bankart repair. Bone Jt. J..

[101] Di Giacomo G., Peebles L.A., Pugliese M., Dekker T.J., Golijanin P., Sanchez A., Provencher M.T. (2020). Glenoid track instability management score: Radiographic modification of the instability severity index score. Arthrosc. J. Arthrosc. Relat. Surg..

[102] Griesser M.J., Harris J.D., McCoy B.W., Hussain W.M., Jones M.H., Bishop J.Y., Miniaci A. (2013). Complications and re-operations after Bristow-Latarjet shoulder stabilization: A systematic review. J. Shoulder Elb. Surg..

[103] Moroder P., Schulz E., Wierer G., Auffarth A., Habermeyer P., Resch H., Tauber M. (2019). Latarjet Procedure Versus Iliac-Crest Bone Graft Transfer for Treatment of Anterior Shoulder Instability with Glenoid Bone Loss: A Prospective Randomized Trial. J. Shoulder Elb. Surg..

[104] Kwapisz A., Fitzpatrick K., Cook J.B., Athwal G.S., Tokish J.M. (2018). Distal clavicular osteochondral autograft augmentation for glenoid bone loss: A comparison of radius of restoration versus Latarjet graft. Am. J. Sports Med..

[105] Provencher M.T., Peebles L.A., Aman Z.S., Bernhardson A.S., Murphy C.P., Sanchez A., Dekker T.J., LaPrade R.F., Di Giacomo G. (2019). Management of the failed Latarjet procedure: Outcomes of revision surgery with fresh distal tibial allograft. Am. J. Sports Med..

[106] Frank R.M., Romeo A.A., Richardson C., Sumner S., Verma N.N., Cole B.J., Nicholson G.P., Provencher M.T. (2018). Outcomes of Latarjet versus distal tibia allograft for anterior shoulder instability repair: A matched cohort analysis. Am. J. Sports Med..

[107] Bonazza N.A., Riboh J.C. (2020). Management of Recurrent Anterior Shoulder Instability after Surgical Stabilization in Children and Adolescents. Curr. Rev. Musculoskelet. Med..

[108] Kuroda S., Sumiyoshi T., Moriishi J., Maruta K., Ishige N. (2001). The natural course of atraumatic shoulder instability. J. Shoulder Elb. Surg..

[109] Misamore G.W., Sallay P.I., Didelot W. (2005). A longitudinal study of patients with multidirectional instability of the shoulder with seven- to ten-year follow-up. J. Shoulder Elb. Surg..

[110] Kłaptocz P., Solecki W., Grzegorzewski A., Błasiak A., Brzóska R. (2021). Effectiveness of conservative treatment of multidirectional instability of the shoulder joint. Literature review and meta-analysis. Pol. Prz. Chir..

[111] Watson L., Balster S., Lenssen R., Hoy G., Pizzari T. (2018). The effects of a conservative rehabilitation program for multidirectional instability of the shoulder. J. Shoulder Elb. Surg..

[112] Chu J.C., Kane E.J., Arnold B.L., Gansneder B.M. (2002). The effect of a neoprene shoulder stabilizer on active joint-reposition sense in subjects with stable and unstable shoulders. J. Athl. Train..

[113] Williams S., Whatman C., Hume P.A., Sheerin K. (2012). Kinesio taping in treatment and prevention of sports injuries: A meta-analysis of the evidence for its effectiveness. Sports Med..

[114] Ide J., Maeda S., Yamaga M., Morisawa K., Takagi K. (2003). Shoulder-strengthening exercise with an orthosis for multidirectional shoulder instability: Quantitative evaluation of rotational shoulder strength before and after the exercise program. J. Shoulder Elb. Surg..

[115] Barrett C. (2015). The clinical physiotherapy assessment of non-traumatic shoulder instability. Shoulder Elb..

[116] Koyuncu E., Nakipoğlu-Yüzer G.F., Doğan A., Özgirgin N. (2010). The effectiveness of functional electrical stimulation for the treatment of shoulder subluxation and shoulder pain in hemiplegic patients: A randomized controlled trial. Disabil. Rehabil..

[117] Kiss J., Damrel D., Mackie A., Neumann L., Wallace W. (2001). Non-operative treatment of multidirectional shoulder instability. Int. Orthop..

[118] Vavken P., Tepolt F.A., Kocher M.S. (2016). Open inferior capsular shift for multidirectional shoulder instability in adolescents with generalized ligamentous hyperlaxity or Ehlers-Danlos syndrome. J. Shoulder Elb. Surg..

[119] Rolfes K. (2015). Arthroscopic treatment of shoulder instability: A systematic review of capsular plication versus thermal capsulorrhaphy. J. Athl. Train..

[120] Witney-Lagen C., Hassan A., Doodson A., Venkateswaran B. (2017). Arthroscopic plication for multidirectional instability: 50 patients with a minimum of 2 years of follow-up. J Shoulder Elb. Surg.

[121] Arenas-Miquelez A., Karargyris O., Zumstein M. (2019). All-Arthroscopic, 270° Reconstruction of the Inferior Glenohumeral Ligament With Palmaris Longus Autograft. Arthrosc. Tech..

[122] Bouaicha S., Moor B.K. (2013). Arthroscopic autograft reconstruction of the inferior glenohumeral ligament: Exploration of technical feasibility in cadaveric shoulder specimens. Int. J. Shoulder Surg..

[123] De Carli A., Vadalà A.P., Fedeli G., Scrivano M., Gaj E., Ferretti A. (2021). Anterior Capsulolabral Reconstruction with Semitendinosus Autograft after Latarjet Failure: A Case Report. J. Orthop. Case Rep..

[124] Braun S., Horan M., Millett P. (2011). Open reconstruction of the anterior glenohumeral capsulolabral structures with tendon allograft in chronic shoulder instability. Oper. Orthopädie Traumatol..

[125] Nixon M.F., Stevenson A. (2019). Paediatric shoulder instability. Textbook of Shoulder Surgery.

[126] Rowe C.R., Pierce D.S., Clark J.G. (1973). Voluntary dislocation of the shoulder: A preliminary report on a clinical, electromyographic, and psychiatric study of twenty-six patients. JBJS.

[127] Hawkins R., Koppert G., Johnston G. (1984). Recurrent posterior instability (subluxation) of the shoulder. J. Bone Jt. Surg. Am. Vol..

